# Guanxin Danshen Formulation Protects against Myocardial Ischemia Reperfusion Injury-Induced Left Ventricular Remodeling by Upregulating Estrogen Receptor β

**DOI:** 10.3389/fphar.2017.00777

**Published:** 2017-11-01

**Authors:** Xuehong Deng, Xiaoyan Xing, Guibo Sun, Xudong Xu, Haifeng Wu, Guang Li, Xiaobo Sun

**Affiliations:** ^1^Institute of Medicinal Plant Development, Chinese Academy of Medical Sciences and Peking Union Medical College, Beijing, China; ^2^Beijing Key Laboratory of Innovative Drug Discovery of Traditional Chinese Medicine (Natural Medicine) and Translational Medicine, Beijing, China; ^3^Key Laboratory of Efficacy Evaluation of Chinese Medicine against Glycerolipid Metabolism Disorder Disease, State Administration of Traditional Chinese Medicine, Beijing, China; ^4^Zhongguancun Open Laboratory of the Research and Development of Natural Medicine and Health Products, Beijing, China; ^5^Key Laboratory of Bioactive Substances and Resources Utilization of Chinese Herbal Medicine, Ministry of Education, Beijing, China; ^6^Yunnan Branch, Institute of Medicinal Plant, Chinese Academy of Medical Sciences and Peking Union Medical College, Jinghong, China

**Keywords:** Guanxin Danshen formula, myocardial ischemia reperfusion injury, ventricular remodeling, network pharmacology, estrogen receptor β, PI3K/Akt

## Abstract

**Background:** Guanxin Danshen formulation (GXDSF) is a traditional Chinese herbal recipe recorded in the Chinese Pharmacopeia since 1995 edition, which consists of Salviae miltiorrhizae Radix et Rhizoma, Notoginseng Radix et Rhizoma and Dalbergiae odoriferae Lignum. Our previous research suggested GXDSF had positive effect on cardiovascular disease. Therefore, the aim of this study was to elucidate the effects of GXDSF on myocardial ischemia reperfusion injury-induced left ventricular remodelling (MIRI-LVR).

**Methods:** The effects of GXDSF on cardiac function were detected by haemodynamics and echocardiograms. The effects of GXDSF on biochemical parameters (AST, LDH and CK-MB) were analyzed. Histopathologic examinations were performed to evaluate the effect of GXDSF on cardiac structure. In addition, the Traditional Chinese Medicine Systems Pharmacology (TCMSP) database was used to predict the main target of GXDSF. Target validation was conducted by using western blots and immunofluorescent double staining assays.

**Results:** We found that +dp/dt and LVSP were significantly elevated in the GXDSF-treated groups compared with the MIRI-LVR model group. Left ventricular ejection fraction (LVEF) and left ventricular fractional shortening (LVFS) were increased in the GXDSF-treated groups compared with the model group. All biochemical parameters (AST, LDH and CK-MB) were considerably decreased in the GXDSF-treated groups compared with the model group. Fibrosis parameters (collagen I and III, α-SMA, and left ventricular fibrosis percentage) were decreased to different degrees in the GXDSF-treated groups compared with the model group, and the collagen III/I ratio was elevated by the same treatments. TCMSP database prediction and western blot results indicated that estrogen receptor β (ERβ) could be the main target of GXDSF. PHTPP, a selective antagonist of ERβ, could inhibit the expression of ERβ and the phosphorylation of PI3K and Akt in myocardial tissue induced by GXDSF, and partly normalize the improving effects of GXDSF on +dp/dt, LVEF, LVFS, LDH, CK-MB, α-SMA and myocardial fibrosis.

**Conclusion:** Collectively, GXDSF showed therapeutic potential for use in the prevention and treatment of myocardial ischemia reperfusion injury-induced ventricular remodeling by upregulating ERβ via PI3K/Akt signaling. Moreover, these findings may be valuable in understand the mechanism of disease and provide a potential therapy of MIRI-IVR.

## Introduction

Ischemia heart disease (IHD) was the leading cause of death worldwide. In 2013, the death toll rose to 8.1 million (Shepard et al., 2015). After an acute myocardial infarction, thrombolytic therapy or primary percutaneous coronary intervention (PCI) are the most commonly selected to reduce the size of the myocardial infarct in clinical treatment. However, the proceed of restoring blood flow can induce lethal reperfusion injury and lead to left ventricular remodeling which is the most critical reason why the frequent occurrence of heart failure after an acute myocardial infarction and the rate of death after an acute myocardial infarction approaches 10% (Yellon and Hausenloy, 2007). Currently, there are no effective and safe therapies for preventing MIRI-LVR (Hausenloy and Yellon, 2013), which prompted us to explore some promising approaches.

Guanxin Danshen formula (GXDSF) is a traditional Chinese herbal recipe recorded in the Chinese Pharmacopeia since 1995 edition, which consists of Salviae miltiorrhizae Radix et Rhizoma (derived from Lamiaceae *Salvia miltiorrhiza* Bunge), which exerts the effects of activating blood and removing stasis, Notoginseng Radix et Rhizoma (derived from Araliaceae *Panax notoginseng* (Burkill) F.H.Chen), which exerts the effects of scattered stasis and hemostasis, and Dalbergiae odoriferae Lignum (derived from Leguminosae *Dalbergia odorifera* T.C.Chen), which exerts the effects of regulating qi and relieving pain. The literature data indicated that some components of GXDSF have a definite protection on cardiovascular disease (CVD). For example, Tanshinone IIA could inhibit the differentiation of myocardial fibroblasts and against MIRI (Mao et al., 2016; Li et al., 2016). Cryptotanshinone is able to attenuate cardiac fibrosis induced by isoprenaline (Ma et al., 2012). In addition, a combination of Salvianolic acid B and ginsenoside Rg1 showed significant effects on down-regulation of myocardial infarct size, maintenance of myocardium structure, improvement on cardiac function in rats with I/R injury (Deng et al., 2015). While, as a Chinese traditional formula, only few studies (Zhao et al., 2006; Dou and Sun, 2015; Dou et al., 2015) have explained the cardio-protection of GXDSF against MIRI. No preclinical or clinical data have shown that whether GXDSF could interfere the ventricular remodeling development induced by MIRI or not. Furthermore, there are always some limitations on effective and safety problems induced by drugs applied in clinical applications, and traditional Chinese formula could be a huge promising medication for CVD. Therefore, it could be very significance to interpret the mechanism of GXDSF in the cardiac protection against MIRI-LVR.

In this study, we first used the Traditional Chinese Medicine Systems Pharmacology (TCMSP) database to build a compound-target-disease network and find out the hub-protein. Then we systematically explored the protective effect of GXDSF in a classical animal model by versatile pharmacological assays. At last, we verified the hub-protein by molecular expression and loss-of-function tests to preliminarily elucidate the protective effect of GXDSF on MIRI-LVR and its mechanism of action.

## Materials and Methods

### Reagents and Materials

GXDSF consists of Salviae miltiorrhizae Radix et Rhizoma (#20141201), Notoginseng Radix et Rhizoma (#20150102) and Dalbergiae odoriferae Lignum (#20140801), was provided by Zhongfa Industrial and Commercial Group Yerui Pharmaceutical Co., Ltd. (Heilongjiang, China). The oil extract of Dalbergiae odoriferae Lignum was determined to be over 1% in accordance with the standard of the Chinese Pharmacopeia 2015.

Aspartate transaminase (AST), lactate dehydrogenase (LDH) and creatine kinase-MB (CK-MB) assay kits were obtained from BioSino Biotechnology & Science, Inc. (Beijing, China). Antibodies for collagen I (ab21287, 1:500), collagen III (ab7778, 1:1000), α–tubulin (ab176560, 1:5000), ERα (ab16460, 1:1000), ERβ (ab16813, 1:1000) and α-smooth muscle actin (α-SMA, ab15734, 1:800) were obtained from Abcam (Cambridge, MA, United States). Immunofluorescent double staining antibodies including α-SMA (ab32575, 1:200), ERβ (ab212351, 1:1000), donkey anti-mouse IgG H&L (ab150109, 1:400) and donkey anti-rabbit IgG H&L (ab150064, 1:400) were also obtained from Abcam (Cambridge, MA, United States). PI3K (sc-67306, 1:200), p-PI3K (sc-293115, 1:200), Akt (sc-8312, 1:100), and p-Akt (sc-271966, 1:100) antibodies were purchased from Santa Cruz Biotechnology, Inc. (Santa Cruz, CA, United States). 4-[2-Phenyl-5,7-bis(trifluoromethyl)pyrazolo[1,5-a]pyrimidin-3-yl]-phenol (PHTPP) was obtained from Tocris Bioscience (R&D Systems, United States). Gentamicin sulfate (#H41020249) was purchased from Xinxiang Dongsheng Pharmaceutical Co., Ltd. (Henan, China).

The present study required the use of an ALC-V8S animal respirator (Shanghai Alcott Biotech Co., Ltd., China), an ALC-HTP thermal insulation blanket (Shanghai Alcott Biotech Co., Ltd., China), AD Instruments for multi-channel electrophysiological recordings (BIOPAC, United States), a Germinator 500 to dry sterilize surgical instruments in seconds (Harvard Apparatus, United States), and a Vevo 770 high-resolution imaging system with a high frequency probe (40–60 MHz, VisualSonics; Canada).

### Drug Preparation and Administration

To prepare GXDSF, Salviae miltiorrhizae Radix et Rhizoma (200 g), Notoginseng Radix et Rhizoma (200 g) and the oil extract of Dalbergiae odoriferae Lignum (1.75 mL) were mixed as described in the Chinese Pharmacopoeia 2015. The contents of representative chemical compositions in GXDSF were determined by HPLC. Notoginsenoside R1, ginsenoside Rg1, salvianolic acid B, ginsenoside Rb1, cryptotanshinone, tanshinone I and tanshinone IIA were purchased from the National Institute for Food and Drug Control. All of the purities were above 98% by HPLC analysis. The data were obtained using Waters 2695 Series HPLC with DAD. A Tnature C_18_ reserved-phase column (4.6 mm × 250 mm; 5 μm; DataApex and ACCHROM Instrument Technologies Co. Ltd, Ireland) was used. The sample injection volume was 10 μL. The mobile phase consisted of 0.1% phosphoric acid (A) and acetonitrile (B) with gradient elution and the flow rate was 1.0 mL/min. The gradient program (A/B, v/v) was as follows: 78:22 (*t* = 0 min), 78:22 (*t* = 20 min), 40:60 (*t* = 45 min), 40:60 (*t* = 70 min), 22:78 (*t* = 80 min). The detection wavelength was set at 203 nm (notoginsenoside R1, ginsenoside Rg1 and ginsenoside Rb1), 270 nm (cryptotanshinone, tanshinone I and tanshinone IIA) and 286 nm (salvianolic acid B). The column temperature was set to 30°C. The HPLC chromatogram was shown in Supplementary Figures 1 and 2. The contents of notoginsenoside R1 (2.34%), ginsenoside Rg1 (9.51%), salvianolic acid B (0.50%), ginsenoside Rb1 (8.63%), cryptotanshinone (0.84%), tanshinone I (0.55%) and tanshinone IIA (1.71%) in GXDSF extracts were determined.

GXDSF were dissolved in a 0.5% aseptic sodium carboxymethylcellulose (CMC-Na) solution. Twenty-four hours after successful surgery, GXDSF was given to rats by gavage for 14 consecutive days. Similarity, estradiol was dissolved in phosphate-buffered saline (PBS, containing 1% anhydrous ethanol; 10 μg/mL) and given by intraperitoneal injection (10 mL/kg) for 14 consecutive days. PHTPP was dissolved in PBS (300 μg/mL; containing 4% DMSO and 46% anhydrous ethanol) and administered by subcutaneous injection for 15 consecutive days after successful surgery.

### TCMSP Application

We explored the chemical components of each medical plant in GXDSF using the TCMSP database and screened the active constituents according to an *in silico* ADME principle: oral bioavailability (OB) > 30% and drug-likeness (DL) > 0.18. Then, we used these active ingredients as bait to fish for related targets and diseases. The component-target-disease network was constructed using Cytoscape 3.4.0.

### Animals

A total of 130 male specific pathogen-free (SPF) Sprague-Dawley (SD) rats (aged 7–8 weeks; weighing 300–330 g) were purchased from Beijing Vital River Laboratory Animal Technology Co., Ltd. (Beijing, China), used after a week of acclimatization. Five animals were allocated per polycarbonate cage, and the housing condition was in accordance with the national standard of the People’s Republic of China (GB14925-2010). The animals were acclimated in standard laboratory conditions (ventilated room, 25 ± 1°C, 60% humidity, 12 h light/dark cycle) and had free access to standard water and food. All experimental procedures and protocols were conducted in accordance with the American Physiological Society “Guiding Principles in the Care and Use of Animals” and were approved by the Laboratory Animal Ethics Committee of the Institute of Medicinal Plant Development, Peking Union Medical College (SLXD-20151214).

### Surgical Procedures

The male SD rats were anesthetized with an intraperitoneal injection of sodium pentobarbital (40 mg/kg). The electrocardiograph (ECG) was recorded with a standard limb lead II using a multi-channel electrophysiological recording instrument through the whole surgical experiment. After tracheal cannulation, the rats were ventilated with room air using the ALC-V8S animal respirator (tidal volume = 1 mL; respiratory ratio = 1:1; 70 breaths/min). Thoracotomy was conducted between the 3rd and 4th intercostal spaces, and after excising the pericardium, the heart was exposed entirely. A 6-0 silk suture was passed beneath the left auricle by approximately 2 mm. After a 5-min stabilization period, a small soft tube (∼35 mm) was passed over both ends of the suture to occlude the left anterior descending coronary artery. All animals were subjected to a 30-min ischemia period with the ligature and a 14-day reperfusion period with drug administration. At the same time, the rats were given gentamicin sulfate (1.5 mL/kg) for 3 days to avoid wound infection. The ischemia zone (left ventricular myocardial tissues below the ligature) were investigated for further experiments. All procedures were conducted in accordance with the American Physiological Society “Guiding Principles in the Care and Use of Animals.”

### Experimental Design

#### Part 1

Sixty male SD rats were randomly divided into six groups: Sham group (negative group, *n* = 10), MIRI-LVR model group (*n* = 10), Simvastatin group (positive group, 5 mg/kg, i.g., *n* = 10), low-dose GXDSF group (0.648 g/kg Salviae miltiorrhizae Radix et Rhizoma, 0.648 g/kg Notoginseng Radix et Rhizoma and 5.67 μL/kg the oil extract of Dalbergiae odoriferae Lignum, which is 4-fold higher than the exposure in humans at the recommended clinical dose, i.g., *n* = 10), moderate-dose GXDSF group (1.296 g/kg Salviae miltiorrhizae Radix et Rhizoma, 1.296 g/kg Notoginseng Radix et Rhizoma and 11.34 μL/kg the oil extract of Dalbergiae odoriferae Lignum, which is 8-fold higher than the exposure in humans at the recommended clinical dose, i.g., *n* = 10), and high-dose of GXDSF treated group (2.592 g/kg Salviae miltiorrhizae Radix et Rhizoma, 2.592 g/kg Notoginseng Radix et Rhizoma and 22.68 μL/kg the oil extract of Dalbergiae odoriferae Lignum, which is 16-fold higher than the exposure in humans at the recommended clinical dose, i.g., *n* = 10).

#### Part 2

Seventy male SD rats were randomly allocated into seven groups: Sham group (negative group, *n* = 10), MIRI-LVR model group (*n* = 10), estradiol group (positive group, 100 μg/kg, i.p., *n* = 10), high-dose of GXDSF treated group (2.592 g/kg Salviae miltiorrhizae Radix et Rhizoma, 2.592 g/kg Notoginseng Radix et Rhizoma and 22.68 μL/kg the oil extract of Dalbergiae odoriferae Lignum, which is 16-fold higher than the exposure in humans at the recommended clinical dose, i.g., *n* = 10), PHTPP (150 μg/kg, s.c.) + high-dose of GXDSF treated group (i.g., *n* = 10), PHTPP group (150 μg/kg, s.c., *n* = 10), and normal animals treated with high-dose of GXDSF treated group (i.g., *n* = 10).

### Hemodynamics Determination

Hemodynamic parameters were measured in lightly anesthetized rats (sodium pentobarbital, i.p., 40 mg/kg) 2 h after the last GXDSF treatment (Part 1 and Part 2). A polyethylene catheter was inserted via the right carotid artery and connected to the AD Instrument for multi-channel electrophysiological recording (BIOPAC, United States) to measure left ventricular end-diastolic pressure (LVEDP), left ventricular systolic pressure (LVSP), and the maximal rate of rise and decrease of left ventricular pressure (±dp/dt).

### Echocardiography

Rats were safely and rapidly anesthetized with isoflurane, and echocardiography was conducted 2 h after the last GXDSF treatment. Echocardiograms were generated using the Vevo 770 high-resolution imaging system. M-mode tracing of the left ventricle was obtained from the parasternal long-axis view to calculate LVEF (%) and LVFS (%).

### Biochemical Parameter Measurements

At the end of drug administration, the animals were intraperitoneally anesthetized with sodium pentobarbital (40 mg/kg), and blood samples were collected from the abdominal aorta into EDTA tubes. Then, plasma samples were obtained by centrifugation at 3000 ×*g* for 10 min at 4°C. AST, LDH and CK-MB were measured using an AU480 automatic biochemistry analyser (Beckman Coulter, Inc., United States) followed the kit instructions.

### Histopathologic Examination

After the preparation of plasma samples, the heart was excised, and the wet weight was obtained. Four of ten tissues in each group (4/10) were fixed in the 10% neutral formalin liquid for histopathologic analysis. And the remaining tissues (6/10) were stored in liquid nitrogen for western blotting analysis. After standard paraffin embedding, 5-μm thick sections were obtained and stained with Masson’s trichrome. Histopathologic images were acquired using a light microscope and analyzed using Image-Pro Plus software (IPP 6.0, United States). The fibrosis area and left ventricular area were measured, and the fibrosis ratio was calculated as the fibrosis area divided by the total left ventricular area. The ratio of each tissue was calculated, and the mean ratio was obtained as the fibrosis ratio of each group.

### Immunohistological Assay

Neutral formalin liquid-fixed, paraffin-embedded tissues were sectioned at a thickness of 5 μm for immunostaining using a secondary poly-HRP anti-rabbit IgG antibody (ZSGB-BIO Co, PV-9001, China). The sections were deparaffinized in xylene and hydrated in an ethanol gradient. Antigen retrieval was performed by heating the sections in a microwave oven, followed by the blocking of endogenous peroxide activity with hydrogen peroxide (H_2_O_2_) for 15 min. Then, the sections were washed three times with phosphate-buffered saline (PBS) and blocked with 10% normal goat serum (ZSGB-BIO Co, ZLI-9022, China) for 20 min. After that, the tissues were incubated with collagen I (Abcam, ab21287) and collagen III (Abcam, ab7778) antibodies in the antibody dilution solution (ZSGB-BIO Co, ZLI-9030, China) overnight at 4°C. As a negative control, the primary antibody was replaced with antibody dilution solution. As a positive control, we used rat skin tissue instead of heart tissue. After subsequent washing with PBS, poly-HRP anti-rabbit IgG was used as a secondary antibody. Colorization was achieved by applying diaminobenzidine (DAB Kit) (ZSGB-BIO Co, ZLI-9019, China) with a haematoxylin counterstain. Sections were dehydrated in ethanol before mounting coverslips.

The content of collagen I and III was estimated from 5 randomly selected fields at a magnification of 20×. Images were acquired on an Olympus DP70 Microscope Digital Camera (Olympus, Inc., Tokyo, Japan). The densities of collagen I and III were measured using Image-Pro Plus software (IPP 6.0, United States), and the data are presented as the ratio of collagen I- or III-labeled area to total area.

### Western Blotting

Samples from the ischemia zone were homogenized 1:10 (wt:vol, g/mL) using tissue protein extraction kit (CWBIO, CW0891M, China) supplemented with 1% phosphatase inhibitor cocktail (CWBIO, CW2383S, China) and centrifuged at 12000 ×*g* for 10 min at 4°C. The protein concentration in the supernatant was determined by BCA assay (CWBIO, CW0014, China). Protein extracts (3.5 mg/mL) diluted with 5× SDS loading buffer (CWBIO, CW0045S, China) were denatured in boiling water for 5 min and separated by 8% SDS-PAGE. Then, the proteins were electrotransferred onto a nitrocellulose (NC) membrane (100 V, 300 mA), which was subsequently rinsed with 5% non-fat dry milk blocking solution for 3 h. Next, the NC membranes were incubated with primary antibodies (anti-α-tubulin, anti-ERα, anti-ERβ, anti-α-SMA, anti-PI3K, anti-p-PI3K, anti-Akt and anti-p-Akt) at 4°C overnight. Then, the NC membrane was washed with TBST (CWBIO, CW0043S, China), incubated with secondary horseradish peroxidase (HRP)-conjugated goat anti-rabbit IgG H&L antibody (Abcam, ab16813, 1:10000) or rabbit anti-mouse IgG H&L antibody (Abcam, ab6728, 1:5000) for 2 h at room temperature, and exposed to enhanced ECL (CWBIO, CW0049M, China) for 5 min. The membrane was then subjected to imaging and analysis using ChemiDoc^TM^ XRS+ with Image Lab^TM^ software (Bio-Rad, Berkeley, CA, United States). The experiment was replicated three times with 6 samples in each group, and the results were presented as the mean ± SD.

### Immunofluorescent Double Staining

Heart tissue paraffin section were deparaffinised and antigen retrieved using the same steps with immunohistological assay. 10% donkey serum with 0.1% Triton-X 100 was used as a blocking buffer. Then, sections were incubated with primary antibodies (ERβ and α-SMA) at 4°C overnight. The sections were washed with PBS for three times and incubated with secondary antibodies (donkey anti-mouse IgG H&L and donkey anti-rabbit IgG H&L) for 1 h at 37°C. After being washed with PBS for three times for 5 min each, sections were mounted with DAPI (ZSGB-BIO Co, ZLI-9557, China). Negative controls had the primary antibody replaced with PBS. All immunostained sections were visualized using a fluorescence microscope (Leica, DM4000B, Germany).

### Statistical Analysis

All data were presented as the mean ± SD of three independent replicates. One-way ANOVA, followed by the Turkey *post hoc* multiple comparison test, was used to analyze differences among groups in SPSS 19.0 software (Cambridge, MA, United States). *P* < 0.05 was considered to indicate statistical significance.

## Results

The experiments in Part 1 and Part 2 were performed as described in the Section “Materials and Methods.” The MIRI-LVR animal model was successfully generated and then utilized in the subsequent experiments.

### Identification of Potential Targets

Importantly, we selected CVD as the target disease to extract a subnetwork, component-target-CVD, using the NetworkAnalyzer plugin in Cytoscape 3.4.0 (Supplementary Figure 3). In accordance with ADME (absorption, distribution, metabolism, and excretion) and drug-likeness screening principles, 59 qualified ingredients and 15 relevant targets were selected from the component-target-CVD network of Salviae miltiorrhizae Radix et Rhizoma. In the same manner, 7 qualified ingredients and 16 relevant targets were selected from the component-target-CVD network of Notoginseng Radix et Rhizoma, and 31 qualified ingredients and 15 relevant targets were selected from the component-target-CVD network of the oil extract of Dalbergiae odoriferae Lignum.

These networks were generated according to degree-based screening of CVD and were reconstructed (Supplementary Figure 4). From the subnetworks, we selected the first five targets, followed by degree ranking of each component from GXDSF (**Table 1**), and we identified ER as the common target with the most degrees of each component from GXDSF. Furthermore, ERβ was determined to potentially play an important role in the protective effect of this formula against CVD.

**Table 1:** Target identification followed by degree-based screening of the three drugs in GXDSF.

Salviae miltiorrhizae Radix et Rhizoma		Notoginseng Radix et Rhizoma		The oil extract of Dalbergiae odoriferae Lignum	
Degree	Target	Degree	Target	Degree	Target
61	ER	7	ER	34	ER
45	ERβ	5	COX-1	31	ERβ
25	COX-1	5	CDPK2	30	COX-1
17	eNOS	3	ERβ	8	eNOS
7	Coagulation factor Xa	3	eNOS	6	Coagulation factor Xa

*ER, Estrogen receptor; ER*β*, Estrogen receptor *β*; COX-1, Cyclooxygenase-1; eNOS, Endothelial nitric oxide synthase*.

### Hemodynamic Changes in MIRI-LVR Rats Treated with GXDSF

#### Part 1

**FIGURE 1:**
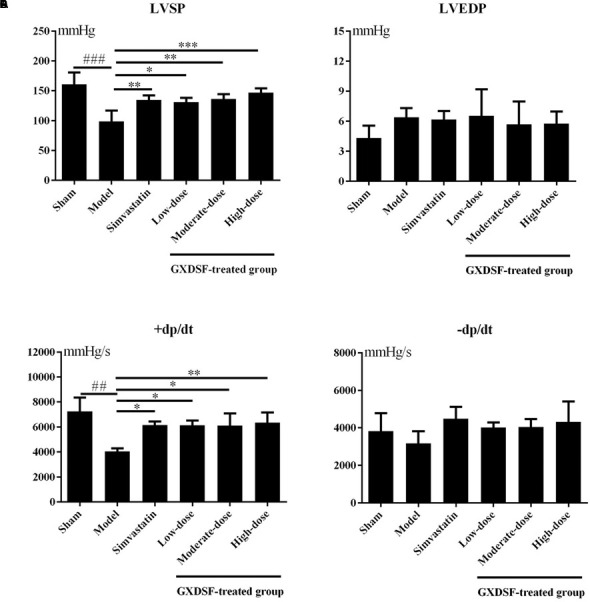
Haemodynamic parameters, including LVSP **(A)**, LVEDP **(B)**, +dp/dt **(C)**, and –dp/dt **(D)**, in MIRI-LVR rats treated with different doses of GXDSF. Compared with the sham group: ^##^*P* < 0.01, ^###^*P* < 0.001. Compared with the MIRI-LVR model group: ^∗^*P* < 0.05, ^∗∗^*P* < 0.01, ^∗∗∗^*P* < 0.001. The data are presented as the mean ± SD (*n* = 10 in each group).

As shown in **Figure 1**, +dp/dt was notably different between the sham group and the MIRI-LVR model group (*P* < 0.01). Among the haemodynamic parameters, +dp/dt was significantly increased in the GXDSF group (low-dose and moderate-dose groups, *P* < 0.05; high-dose group, *P* < 0.01) compared with the MIRI-LVR model group. Furthermore, LVSP was significantly elevated in the simvastatin-treated group (*P* < 0.05) and GXDSF-treated groups in a dose-dependent manner compared with the MIRI-LVR model group (low-dose group, *P* < 0.05; moderate-dose groups, *P* < 0.01; high-dose group, *P* < 0.001).

#### Part 2

**FIGURE 2:**
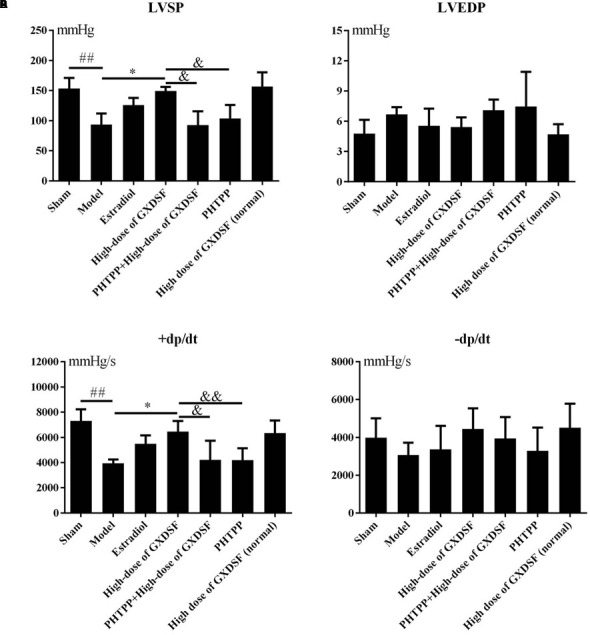
Haemodynamic parameters, including LVSP **(A)**, LVEDP **(B)**, +dp/dt **(C)**, and –dp/dt **(D)**, in MIRI-LVR rats treated with high-dose of GXDSF treated group and PHTPP. Compared with the sham group: ^##^*P* < 0.01. Compared with the MIRI-LVR model group: ^∗^*P* < 0.05. Compared with the high-dose of GXDSF treated group: ^&^*P* < 0.05, ^&&^*P* < 0.01. The data are presented as the mean ± SD (*n* = 10 in each group).

As shown in **Figure 2**, there were no notable differences between the high-dose of GXDSF treated group and the sham group in the normal state. +dp/dt (*P* < 0.05) and LVSP (*P* < 0.05) were significantly decreased in the PHTPP + GXDSF-treated group compared with GXDSF-treated group.

### Echocardiogram Changes in MIRI-LVR Rats Treated with GXDSF

#### Part 1

**FIGURE 3:**
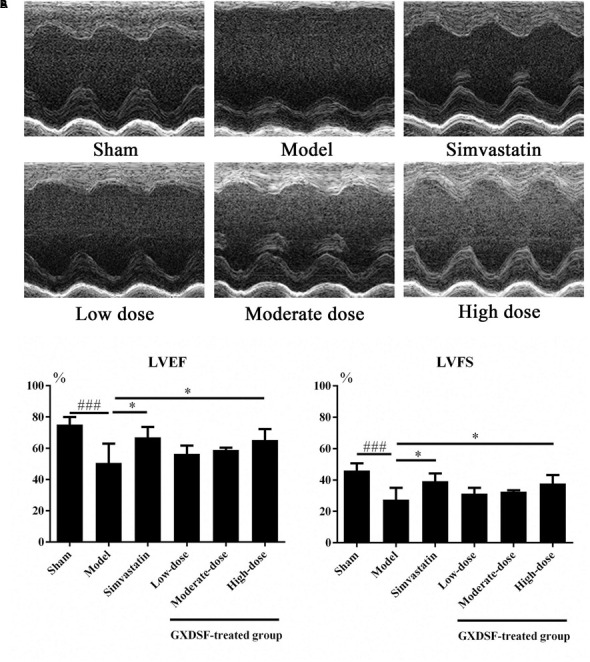
Echocardiogram parameters in MIRI-LVR rats treated with different doses of GXDSF. The echocardiograms **(A)** were obtained using the Vevo 770 high-resolution imaging system. LVEF and LVFS **(B)** data are also shown in bar graphs. Compared with the sham group: ^###^*P* < 0.001. Compared with the MIRI-LVR model group: ^∗^*P* < 0.05. The data are presented as the mean ± SD (*n* = 10 in each group).

As shown in **Figure 3**, left ventricular ejection fraction (LVEF) and left ventricular fractional shortening (LVFS) were notably lower in the MIRI-LVR model group than in the sham group (*P* < 0.001). These two parameters were significantly increased in the high-dose of GXDSF treated group (*P* < 0.05) compared with the MIRI-LVR model group. However, simvastatin treatment did not show any differences compared with the low, moderate and high doses of GXDSF.

#### Part 2

**FIGURE 4:**
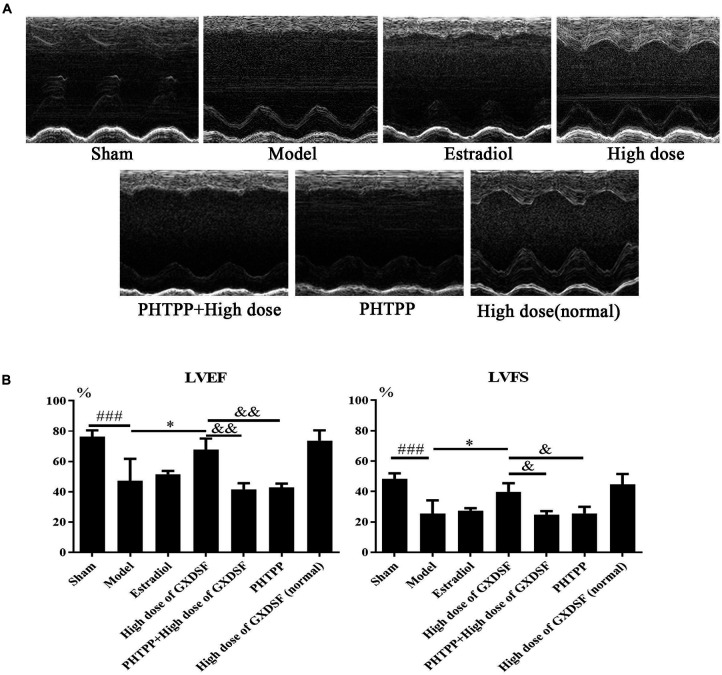
Echocardiogram parameters in MIRI-LVR rats treated with high-dose of GXDSF treated group and PHTPP. The echocardiograms **(A)** were obtained using the Vevo 770 high-resolution imaging system. LVEF and LVFS **(B)** data are also shown in bar graphs. Compared with the sham group, ^###^*P* < 0.001. Compared with the MIRI-LVR model group: ^∗^*P* < 0.01. Compared with the high-dose of GXDSF treated group: ^&^*P* < 0.05, ^&&^*P* < 0.01. The data are presented as the mean ± SD (*n* = 10 in each group).

As shown in **Figure 4**, there were no notable differences in LVEF and LVFS between the high-dose of GXDSF treated group and the sham group in the normal state. The PHTPP + GXDSF group had significantly decreased LVEF (*P* < 0.01) and LVFS (*P* < 0.05) compared with the high-dose of GXDSF treated group. Moreover, the PHTPP-treated group showed no significant differences compared with the model group.

### Effects of GXDSF on Biochemical Parameters in MIRI-LVR Rats

#### Part 1

**FIGURE 5:**
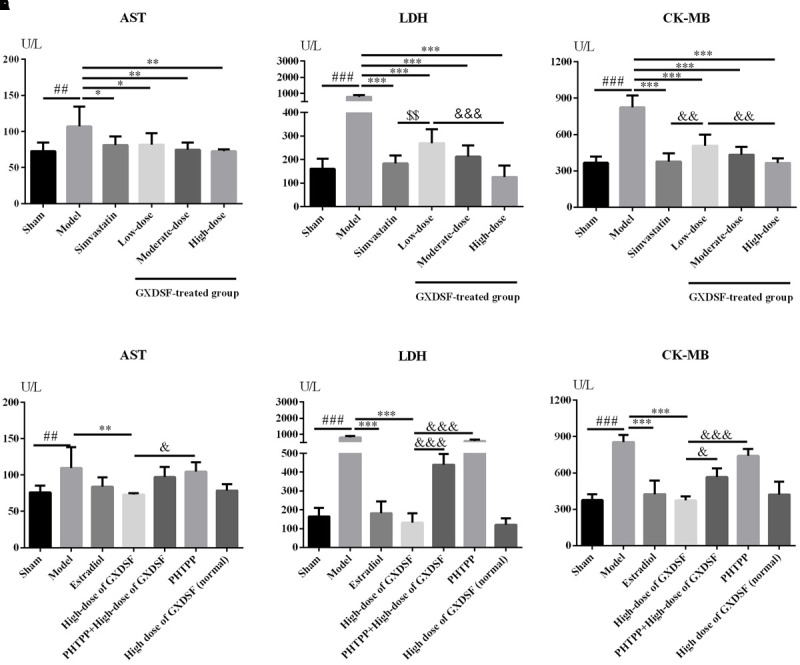
Biochemical parameters, including AST **(A,D)**, LDH **(B,E)** and CK-MB **(C,F)**, in MIRI-LVR rats treated with different doses of GXDSF and PHTPP. Compared with the sham group: ^##^*P* < 0.01, ^###^*P* < 0.001. Compared with the MIRI-LVR model group: ^∗^*P* < 0.05, ^∗∗^*P* < 0.01, ^∗∗∗^*P* < 0.001. Compared with the simvastatin group: ^$$^*P* < 0.01. Compared with the high-dose group: ^&^*P* < 0.05, ^&&^*P* < 0.01, ^&&&^*P* < 0.001. The data are presented as the mean ± SD (*n* = 10 in each group).

As shown in **Figures 5A–C**, all the biochemical parameters (AST, LDH and CK-MB) were increased in the MIRI-LVR model group in comparison with the sham group (AST, *P* < 0.01; LDH and CK-MB, *P* < 0.001). Accordingly, these three parameters were significantly decreased in the GXDSF-treated groups (low-dose group, *P* < 0.05, moderate-dose and high-dose groups, *P* < 0.01 in AST; low-dose, moderate-dose and high-dose groups, *P* < 0.001 in LDH and CK-MB) and in the simvastatin-treated group (AST, *P* < 0.05, LDH and CK-MB, *P* < 0.001) compared with the MIRI-LVR model group.

#### Part 2

As shown in **Figures 5D–F**, there was no significant difference in any biochemical parameters (AST, LDH and CK-MB) evaluated between the high-dose of GXDSF treated group in the normal state and the sham group. The PHTPP+GXDSF treated group showed markedly elevated content of LDH (*P* < 0.001) and CK-MB (*P* < 0.05) in comparison with GXDSF-treated group.

### Effects of GXDSF on Myocardial Fibrosis in MIRI-LVR Rats

#### Part 1

**FIGURE 6:**
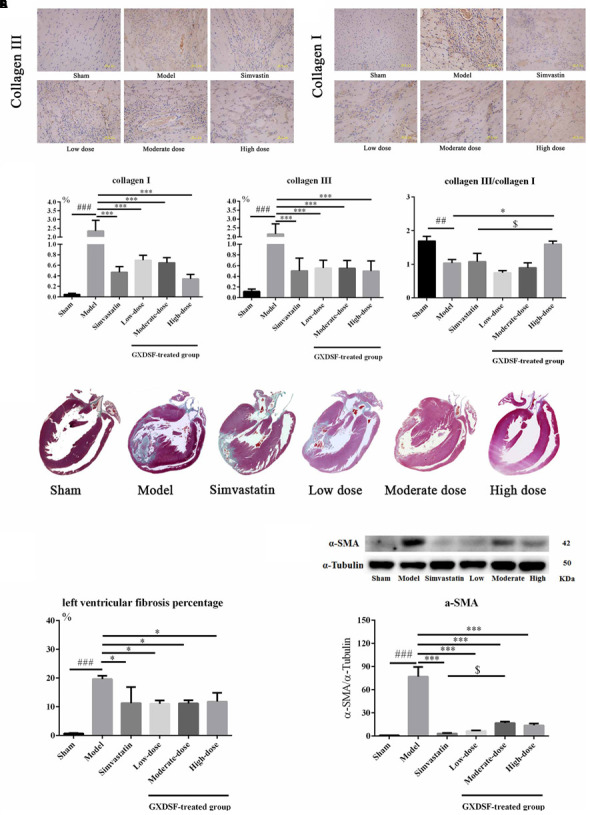
Myocardial fibrosis in MIRI-LVR rats treated with different doses of GXDSF. The immunohistological localization of collagen III **(A)** and collagen I **(B)** is shown. Bars represent 50 μm. The relative expression of collagen I and III and the relative ratio of collagen III/I **(C)** are shown in bar graphs. Masson’s trichrome staining **(D)** and the percentage of left ventricular fibrosis area **(E)** are provided. A gel image with an internal reference (α–tubulin) and the relative expression of α–SMA (**F**; column graph) are shown. Compared with the sham group: ^##^*P* < 0.01, ^###^*P* < 0.001. Compared with the MIRI-LVR model group: ^∗^*P* < 0.05, ^∗∗∗^*P* < 0.001. Compared with the simvastatin group: ^$^*P* < 0.05. The data are presented as the mean ± SD (*n* = 4 in each group).

We explored whether GXDSF had an effect on reversing or ameliorating ventricular fibrosis. As shown in **Figure 6**, the model group showed notable pathological fibrosis compared with the sham group (collagen I, collagen III, α-SMA (α-smooth muscle actin), and left ventricular fibrosis percentage, *P* < 0.001; collagen III/I, *P* < 0.01). Low-, moderate- and high-dose of GXDSF treated group could ameliorate fibrosis by reducing the levels of collagen I (*P* < 0.001), collagen III (*P* < 0.001) and α-SMA (*P* < 0.001) and decreasing the left ventricular fibrosis percentage (*P* < 0.05). High-dose of GXDSF treated group also increased the collage III/I ratio (*P* < 0.05) compared with model group. In addition, GXDSF had stronger positive effects than simvastatin in terms of collage III/I (high dose, *P* < 0.05).

#### Part 2

**FIGURE 7:**
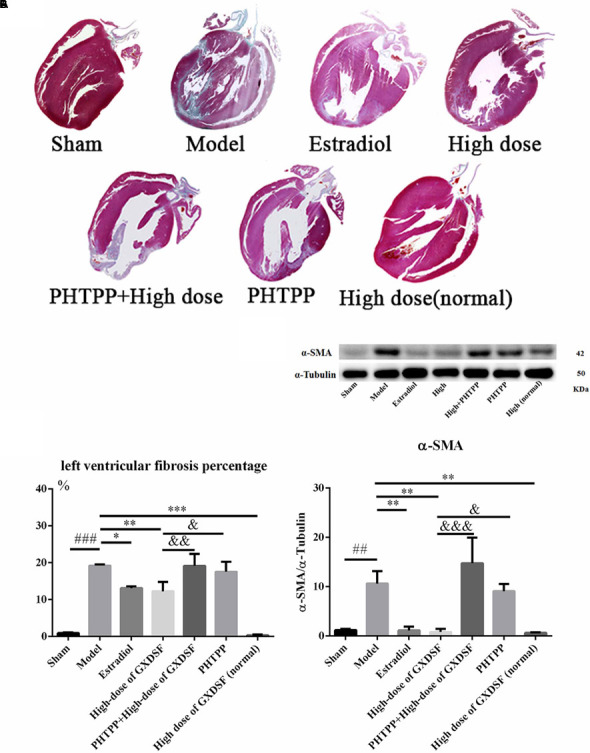
Myocardial fibrosis in MIRI-LVR rats treated with high-dose of GXDSF treated group and PHTPP. Masson’s trichrome staining **(A)** and the percentage of left ventricular fibrosis area **(B)** are shown. A gel image with an internal reference (α–tubulin) and the relative expression of α–SMA (**C**; column graph) are shown. Compared with the sham group: ^##^*P* < 0.01, ^###^*P* < 0.001. Compared with the MIRI-LVR model group: ^∗^*P* < 0.05, ^∗∗^*P* < 0.01, ^∗∗∗^*P* < 0.001. Compared with the high-dose of GXDSF treated group: ^&^*P* < 0.05, ^&&^*P* < 0.01, ^&&&^*P* < 0.001. The data are presented as the mean ± SD (*n* = 4 in each group).

As shown in **Figure 7**, PHTPP could depress the positive effect of GXDSF on myocardial fibrosis (α-SMA, *P* < 0.001; left ventricular fibrosis percentage, *P* < 0.01).

### Effects of GXDSF on the Expression of ER and Associated Proteins in MIRI-LVR Rats

#### Part 1

**FIGURE 8:**
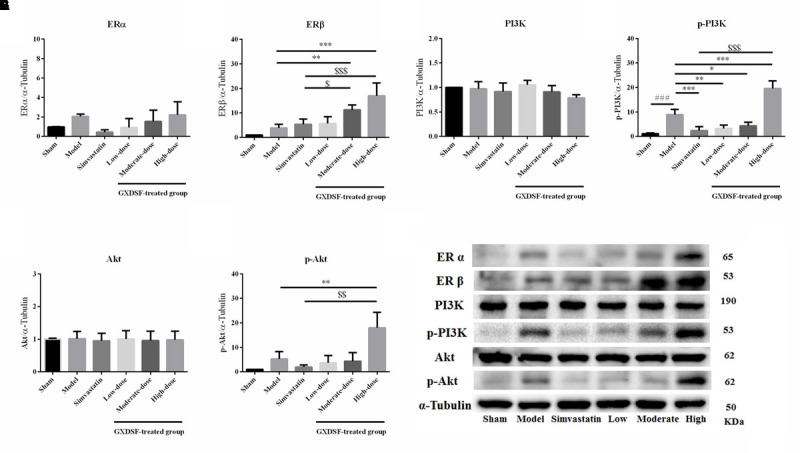
Relative expression of ERα **(A)**, ERβ **(B)**, PI3K **(C)**, p-PI3K **(D)**, Akt **(E)**, p-Akt **(F)** and an internal reference (α–tubulin) **(G)** in MIRI-LVR rats treated with different doses of GXDSF. Compared with the sham group: ^###^*P* < 0.001. Compared with the MIRI-LVR model group: ^∗^*P* < 0.05, ^∗∗^*P* < 0.01, ^∗∗∗^*P* < 0.001. Compared with the simvastatin group: ^$^*P* < 0.05, ^$$^*P* < 0.01, ^$$$^*P* < 0.001. The data are presented as the mean ± SD (*n* = 6 in each group).

Given that ERs might be the effective targets of GXDSF, we evaluated ERα, ERβ and associated proteins (PI3K/Akt) in relevant signaling pathways in animal experiments. GXDSF had a significant dose-dependent effect on ERβ (moderate dose, *P* < 0.01; high dose, *P* < 0.001) but not on ERα. In addition, the positive effect of GXDSF on ERβ was superior to that of simvastatin (moderate dose, *P* < 0.05; high dose, *P* < 0.001). In the same way, high-dose of GXDSF treated group considerably increased the levels of p-PI3K (*P* < 0.001) and p-AKT (*P* < 0.01) (**Figure 8**).

#### Part 2

**FIGURE 9:**
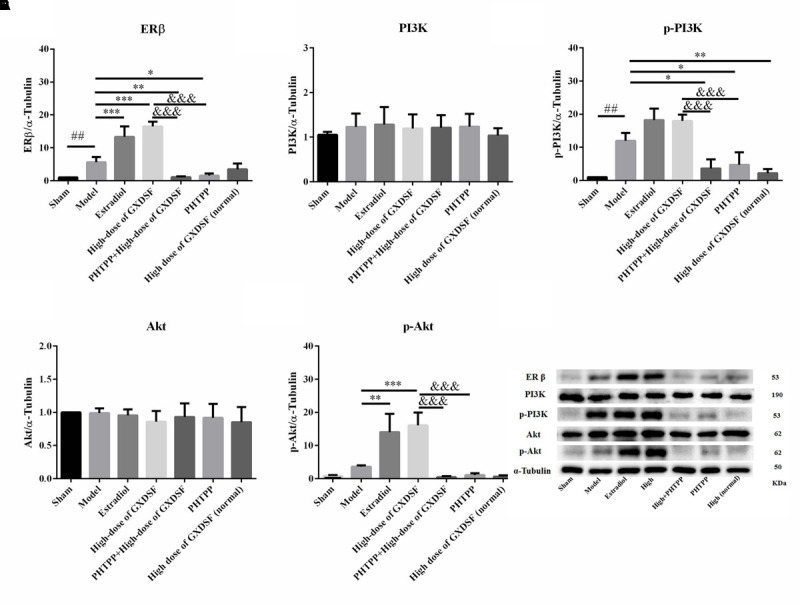
Relative expression of ERβ **(A)**, PI3K **(B)**, p-PI3K **(C)**, Akt **(D)**, p-Akt **(E)** and an internal reference (α–tubulin) **(F)** in MIRI-LVR rats treated with high-dose of GXDSF treated group and PHTPP. Compared with the sham group: ^##^*P* < 0.01. Compared with the MIRI-LVR model group: ^∗^*P* < 0.05, ^∗∗^*P* < 0.01, ^∗∗∗^*P* < 0.001. Compared with the high-dose of GXDSF treated group: ^&&&^*P* < 0.001. The data are presented as the mean ± SD (*n* = 6 in each group).

In the loss-of-function experiment, we investigated whether an ER antagonist could ameliorate the effects on the associated proteins. We found that PHTPP decreased the ERβ expression induced by high-dose of GXDSF treated group and the levels of p-PI3K (*P* < 0.001) and p-Akt (*P* < 0.001) that were stimulated by GXDSF treatment (**Figure 9**).

### Effects of GXDSF on the Distribution of ERβ and α-SMA in MIRI-LVR Rats

**FIGURE 10:**
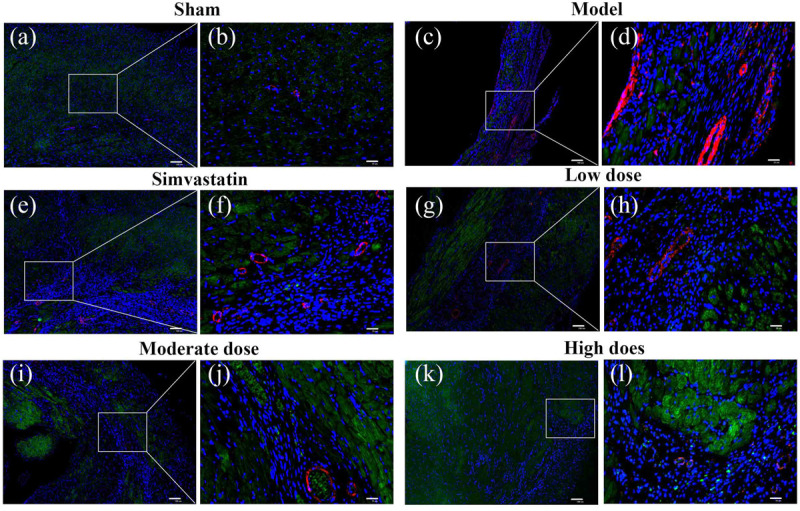
Representative distribution images of ERβ and α-SMA in rats treated with different doses of GXDSF. Immunofluorescence micrographs showing the differences in the distributions of ERβ and α-SMA in the infarct and per-infarct region in all experimental groups **(a,c,e,g,i,k)**. Bars represents 100 μm. The right part of the figures **(b,d,f,h,j,l)** were augmented at 4-fold in the corresponding (the left) part of figures. In addition, the different quasi-colors were depicted for distinguishing ERβ and α-SMA, in which blue represented nuclei, green represented ERβ and red represented α-SMA.

**FIGURE 11:**
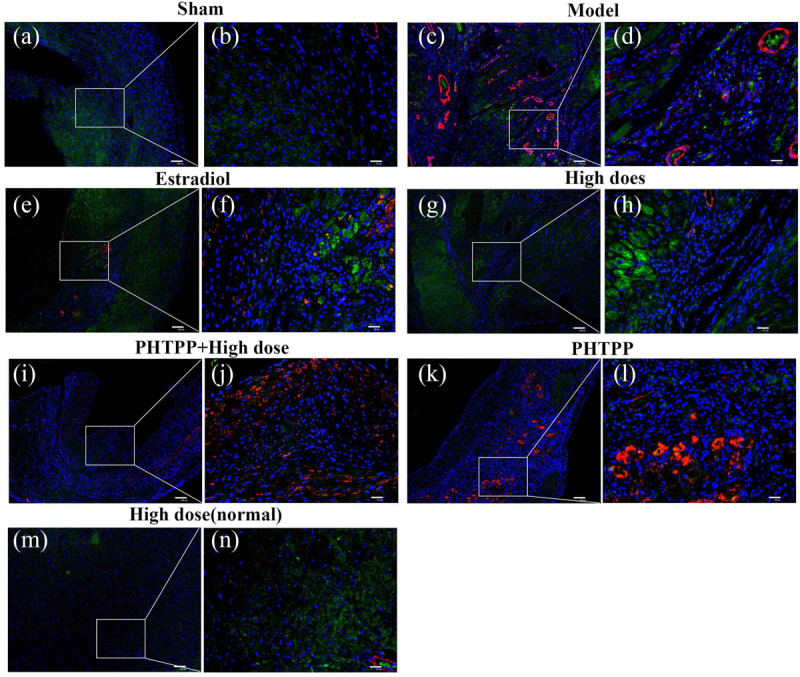
Representative distribution images of ERβ and α-SMA in rats treated with high-dose of GXDSF treated group and PHTPP. Immunofluorescence micrographs showing the differences in the distributions of ERβ and α-SMA in the infarct and per-infarct region in all experimental groups **(a,c,e,g,i,k,m)**. Bars represents 100 μm. The right part of the figures **(b,d,f,h,j,l,n)** were augmented at 4-fold in the corresponding (the left) part of figures. In addition, the different quasi-colors were depicted for distinguishing ERβ and α-SMA, in which blue represented nuclei, green represented ERβ and red represented α-SMA.

Immunofluorescent double staining method was used to assess the distribution of ERβ and α-SMA in the heart of MIRI-LVR rats. As shown in **Figures 10** and **11**, in the sham group, α-SMA was only distributed in the blood vessel wall, and no apparent distribution of ERβ was in the whole heart tissue. The high-dose of GXDSF group in the normal state had a similar situation with the sham group. In the model group, α-SMA were detected throughout the whole infarction region, while ERβ had no apparent distribution in the infarct and peri-infarct region. Low-, moderate-, and high-dose of GXDSF groups could partial offset the distribution of α-SMA in the infarction region, meanwhile, promote the distribution of ERβ in the per-infarct area. In the estradiol group, ERβ was distributed in the peri-infarct area, while a number of α-SMA were still available in the infarction region. PHTPP decreased the ERβ distribution in the peri-infarct area induced by high-dose of GXDSF treated group along with a large amount of α-SMA distribution in the infarct area.

## Discussion

GXDSF was consisted of Salviae miltiorrhizae Radix et Rhizoma (Danshen), which contained mainly two types of constituents, the hydrophilic depsides and lipophilic diterpenoidal quinones (Li et al., 2009), Notoginseng Radix et Rhizoma (Sanqi), which mainly contained saponins, flavonoids and cyclopeptides (Wang T. et al., 2016), and the oil extract of Dalbergiae odoriferae Lignum (Jiangxiang oil). The diversity composition often means variety of pharmacological effects. Of note, Danshen had been applied against a number of diseases and ailments besides CVD, like diabetes (Huang et al., 2015), neurodegeneration (Guo et al., 2015), adipositas (Rahman et al., 2016), hepato- and gastroprotection (Zhou et al., 2015; Li et al., 2016; Wang W. et al., 2016), osteoporosis (Guo et al., 2014), and cancer (Guerram et al., 2015). And varieties of candidate proteins and genes were related with the pharmacological profiles of Danshen. Based on its complexity of composition, traditional uses and wide applications, we chose TCMSP to establish component-target-CVD network. After *in silico* TCMSP-based prediction, we carried out a series of experiments to verify our prediction and explored the preliminary mechanism with *in vivo* molecular biology assays. In summary, we found that GXDSF had a significant protective effect against MIRI-LVR by suppressing fibrosis parameters via the upregulation of ERβ and the PI3K/Akt pathway.

In the haemodynamic assay, systolic function (LVSP and +dp/dt) and diastolic function (LVEDP and -dp/dt) were thoroughly investigated. Only LVSP and +dp/dt were regulated in a dose-dependent manner by GXDSF to reach the normal state, which implied that GXDSF could partly improve systolic function of cardiac ventricles.

In the echocardiograms, LVEF and LVFS were both determined. LVEF is correlated with mortality and represents a cause of death in stable outpatients with heart failure (Curtis et al., 2003). LVFS was determined to have the same prognostic value as LVEF in heart-related diseases by echocardiography (Sun et al., 2015). In our study, LVEF and LVFS were notably elevated by high-dose of GXDSF treated group (*P* < 0.05), while low- and moderate-dose GXDSF did not show any positive effects on these two parameters, which remain to be further studied.

AST, LDH and CK-MB are all biochemical markers for the prediction and diagnosis of ischaemic heart disease, and they were correlated with the heart disease development (Ghormade et al., 2014; Liu et al., 2014). In our experiments, GXDSF treatment significantly decreased all these biochemical markers in accordance with the positive effects of GXDSF administration in terms of protecting against MIRI-LVR.

Collagen subtypes I and III are the predominant interstitial collagens thought to influence cardiac function, and the collagen III/I ratio is considered to be a significant factor (Burgess et al., 1996). However, the collagen III/I ratio plays a very different role in the process of heart disease. The collagen III/I ratio was significantly lower in postoperative atrial fibrillation patients than in control patients in the context of atrial fibrosis (Grammer et al., 2005). However, the collagen III/I ratio was higher in dilated cardiomyopathy patients compared to controls (Soufen et al., 2008). Our results showed that collagens I and III were decreased and that the collagen III/I ratio was increased by high-dose of GXDSF treated group (*P* < 0.05), suggesting that GXDSF has some anti-fibrosis activity. Moreover, α-SMA, which is correlated with myocardial remodeling, is normally expressed in differentiating myocardial fibroblasts and is a biomarker for myocardial hypertrophy in the adult heart (Kern et al., 2013). The left ventricular fibrosis percentage is quantitative measure of myocardial fibrosis in the animal experiments (Tanaka et al., 1986). In our experiments, all the relevant fibrosis parameters were decreased in the GXDSF-treated group, indicating that GXDSF can definitely prevent ventricular fibrosis in the rat heart. In addition, there were no visible differences (**Figures 2**, **4**, **5**, **7**, **9**, **11**) between the sham group and the GXDSF-treated groups in the normal state, which could suggest that GXDSF does not have any beneficial or adverse effects in normal animals.

In our study, TCMSP, which is a network pharmacology-based method, was used to predict targets of GXDSF. Network pharmacology encompasses systems biology, network analysis, connectivity, redundancy anpleiotropy, thus offering a way of thinking about drug discovery and mechanism elucidation in TCM. Hopkins (Hopkins, 2008) first introduced the prospective nature and advantages of network pharmacology in drug discovery. Various studies (Li and Zhang, 2013; Yang et al., 2013; Hao da and Xiao, 2014; Li J.P. et al., 2014; Li S. et al., 2014; Zhang et al., 2014) had shown the power of network analysis in understanding the functional Multiple Components-Multiple Targets-Multiple Pathways pattern of TCM. Based on TCMSP, we predicted that the ER may be the targets of GXDSF. In our experiments, ERα and ERβ were both slightly elevated in the MIRI-LVR model group. Only ERβ was significantly increased in the moderate- and high-dose of GXDSF treated groups, indicating that ERβ is the main target for the cardioprotection by GXDSF. With the increase of expression and distribution of ERβ in myocardium, α-SMA had a decreased distribution in the infarction area. In fact, epidemiologic studies showed that there was a relative increase in ischemic heart disease deaths among men than among woman from 2009 to 2013 (Shepard et al., 2015). Animal studies (Murphy and Steenbergen, 2007; Booth and Lucchesi, 2008) have suggested that ER plays an important role in protection against MIRI. Furthermore, different ER subtypes might not have the same function against MIRI, and the detailed reasons have been broadly discussed (Deschamps et al., 2010). Previous studies suggested that ERα is activated in the acute phase of MIRI and leads to cardioprotection, while chronic activation (14-day) of ERβ leads to cardioprotection, which might support our result that ERβ was the dominant target of GXDSF in the protection against MIRI-LVR.

Collectively, some previous studies have suggested that activation of the PI3K/Akt signaling cascade plays a vital role in ER-mediated acute signaling in cardioprotection against MIRI [ERα (Patten et al., 2004), ERβ (Wang et al., 2009), and GPR30 (Deschamps and Murphy, 2009)]. In addition, ERβ is chronically activated with the upregulation of the NO/SNO signaling pathway, which is involved in cardioprotection (Lin et al., 2009). Besides, Tanshinone IIA inhibited cardiomyocyte hypertrophy, which was mediated through ER, by activating the PI3K/Akt pathway and inhibiting Leu27IGF-II-induced calcineurin and NFATC3 (Weng et al., 2015). Our results showed that the phosphorylated forms of PI3K/Akt were upregulated by GXDSF, which suggests that the PI3K/Akt signaling pathway was activated in response to the chronic upregulation of ERβ by GXDSF.

As a proof-of-mechanism, we verified the target predicted by network pharmacology using the loss-of-function principle by administering a selective ERβ antagonist. However, this method alone is not sufficient, and other evidence, such as from gain-of-function assays, should be obtained. In addition, the role of ERα in ventricular remodeling need to be further elucidated. Furthermore, there are many active ingredients in GXDSF that may play important roles in the prevention of MIRI-LVR. So far, our research team has conducted a series of studies on GXDSF. Based on our preliminary analyses (data not shown), high dose of GXDSF administration could embrace the positive effect against MIRI-IVR, which was considered in our design of experiments. In fact, our team are carrying out research on related work to further clarify the mechanism of dose-dependent effects and effective components of GXDSF on MIRI-LVR.

## Conclusion

On the basis of our experiments and target validation, we inferred that GXDSF had promising protective effects against myocardial ischemia reperfusion injury-induced ventricular remodeling by upregulating ERβ via PI3K/Akt signaling.

## Author Contributions

XS and GS conceived and designed the experiments. XD and XiX carried out the experiments, analyzed the data and wrote the manuscript. XuX and HW conducted the HPLC method for qualification and quantification of representative chemical compositions in GXDSF. GL prepared the figures. All authors reviewed and approved the submitted version of the manuscript.

## Conflict of Interest Statement

The authors declare that the research was conducted in the absence of any commercial or financial relationships that could be construed as a potential conflict of interest.
