# Corrigendum: Guanxin Danshen formulation protects against myocardial ischemia reperfusion injury-induced left ventricular remodeling by upregulating estrogen receptor β

**DOI:** 10.3389/fphar.2024.1488968

**Published:** 2024-10-08

**Authors:** Xuehong Deng, Xiaoyan Xing, Guibo Sun, Xudong Xu, Haifeng Wu, Guang Li, Xiaobo Sun

**Affiliations:** ^1^ Institute of Medicinal Plant Development, Chinese Academy of Medical Sciences and Peking Union Medical College, Beijing, China; ^2^ Beijing Key Laboratory of Innovative Drug Discovery of Traditional Chinese Medicine (Natural Medicine) and Translational Medicine, Beijing, China; ^3^ Key Laboratory of Efficacy Evaluation of Chinese Medicine against Glycerolipid Metabolism Disorder Disease, State Administration of Traditional Chinese Medicine, Beijing, China; ^4^ Zhongguancun Open Laboratory of the Research and Development of Natural Medicine and Health Products, Beijing, China; ^5^ Key Laboratory of Bioactive Substances and Resources Utilization of Chinese Herbal Medicine, Ministry of Education, Beijing, China; ^6^ Yunnan Branch, Institute of Medicinal Plant, Chinese Academy of Medical Sciences and Peking Union Medical College, Jinghong, China

**Keywords:** Guanxin Danshen formula, myocardial ischemia reperfusion injury, ventricular remodeling, network pharmacology, estrogen receptor β, PI3K/Akt

In the published article, there was an error in Figure 4 as published. In Figure 4A, the “Sham” and “High dose” group inadvertently used the same representative images as those in **Figure 3A**. The corrected Figure 4 appears below.

**FIGURE 4:**
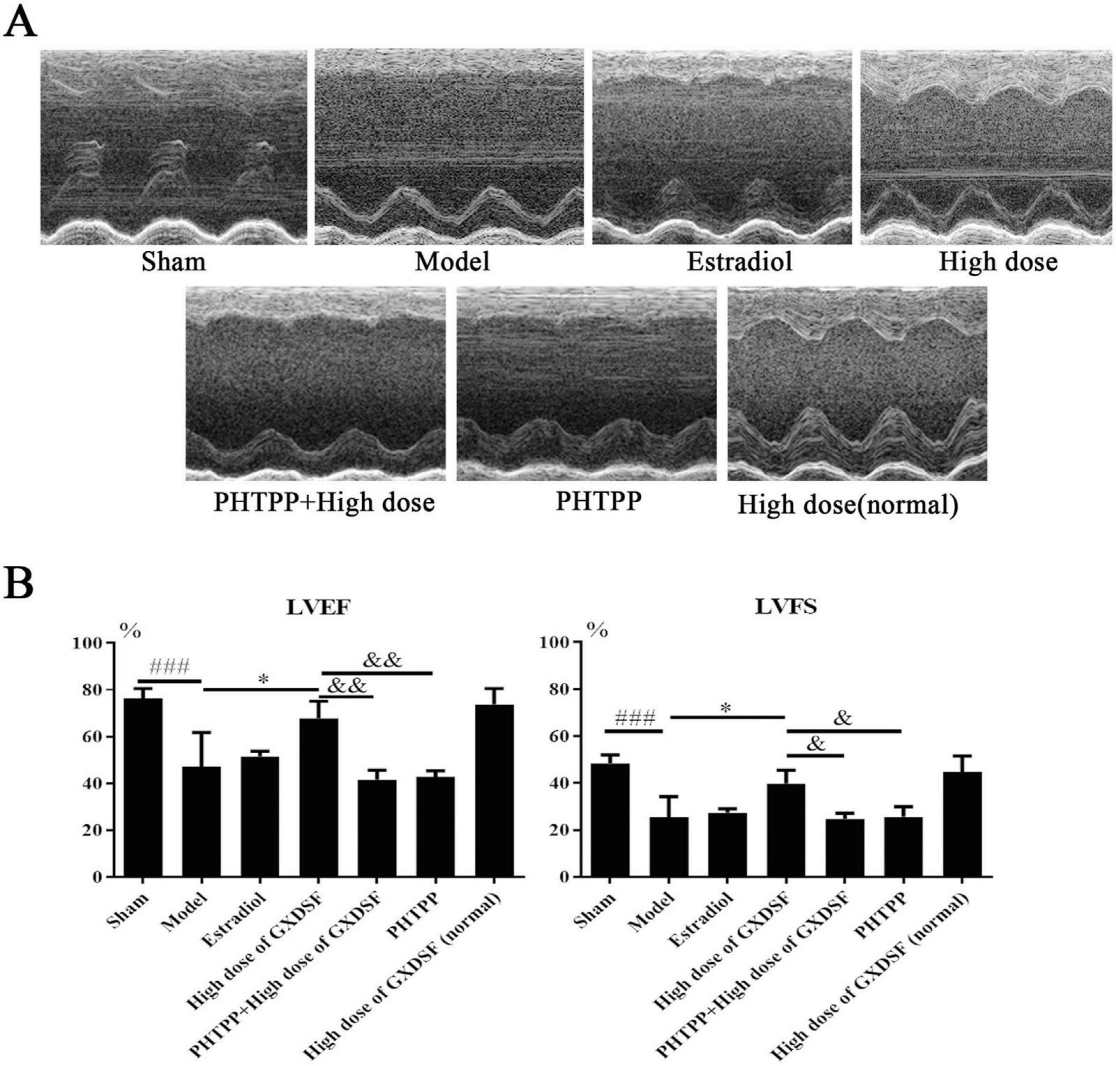
Echocardiogram parameters in MIRI-LVR rats treated with high-dose of GXDSF treated group and PHTPP. The echocardiograms **(A)** were obtained using the Vevo 770 high-resolution imaging system. LVEF and LVFS **(B)** data are also shown in bar graphs. Compared with the sham group, ###*P* < 0.001. Compared with the MIRI-LVR model group: *P < 0.01. Compared with the high-dose of GXDSF treated group: &*P* < 0.05, &&*P* < 0.01. The data are presented as the mean ± SD (n = 10 in each group).

The authors apologize for this error and state that this does not change the scientific conclusions of the article in any way. The original article has been updated.

