# Supplementary material for: Guanxin Danshen Formulation Protects against Myocardial Ischemia Reperfusion Injury-Induced Left Ventricular Remodeling by Upregulating Estrogen Receptor β
Source: Front Pharmacol. 2017 Nov 1;8:777. doi: 10.3389/fphar.2017.00777 (PMC5671976; doi:10.3389/fphar.2017.00777)
Supplement: Supplementary file 1 [file Data_Sheet_1.DOC]

***Supplementary Material***

**Guanxin Danshen formulation protects against myocardial ischemia reperfusion injury-induced left ventricular remodelling by upregulating estrogen receptor β**

**Running title: Cardio-protection of Guanxin Danshen formulation**

**Xuehong Deng^1,2,3,4,5,^**^†^**, Xiaoyan Xing^1,2,3,4,5,^**^†^**, Guibo Sun^1,2,3,4,5,^*, Xudong Xu^1^, Haifeng Wu^1^, Guang Li^1,2,3,4,5,6^, and Xiaobo Sun^1,2,3,4,5,^*^[[1]](#footnote-1)^**

**Page Table of Contents**

**3 Supplementary Figure 1. Typical chromatograms of seven reference compounds in different detection wavelength.**

**4 Supplementary Figure 2. Typical chromatograms of GXDSF in different detection wavelength.**

**5 Supplementary Figure 3. Component-target-CVD networks of Salviae miltiorrhizae Radix et Rhizoma (a), Notoginseng Radix et Rhizoma (b) and the oil extract of Dalbergiae odoriferae Lignum (c).**

**6 Supplementary Figure 4. Degree-based screening networks of Salviae miltiorrhizae Radix et Rhizoma (a), Notoginseng Radix et Rhizoma (b) and the oil extract of Dalbergiae odoriferae Lignum (c).**


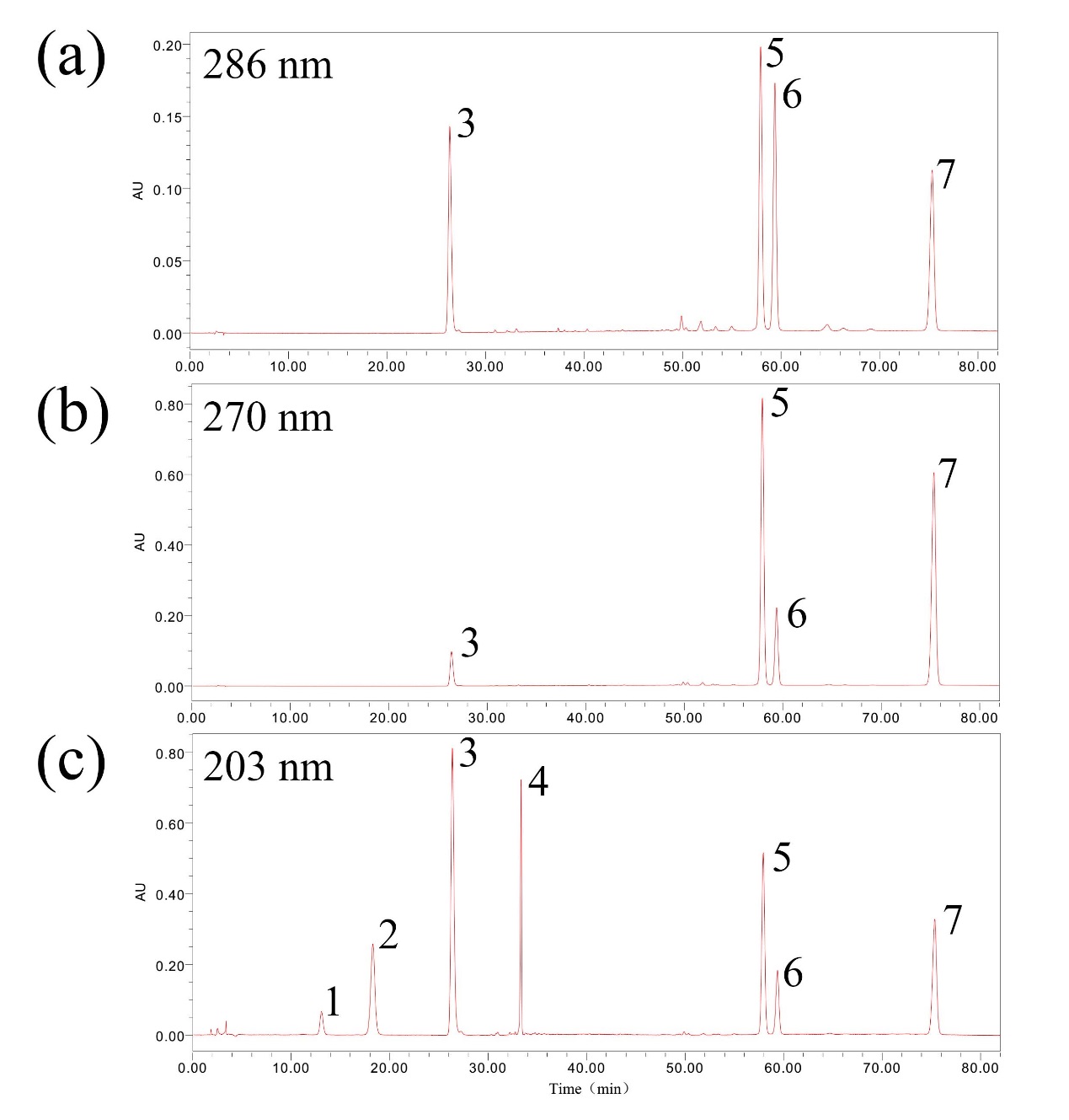


**Supplementary Figure 1. Typical chromatograms of seven reference compounds in different detection wavelength.** 1, notoginsenoside R1; 2, ginsenoside Rg1; 3, salvianolic acid B; 4, ginsenoside Rb1; 5, cryptotanshinone; 6, tanshinone I; 7, tanshinone IIA.


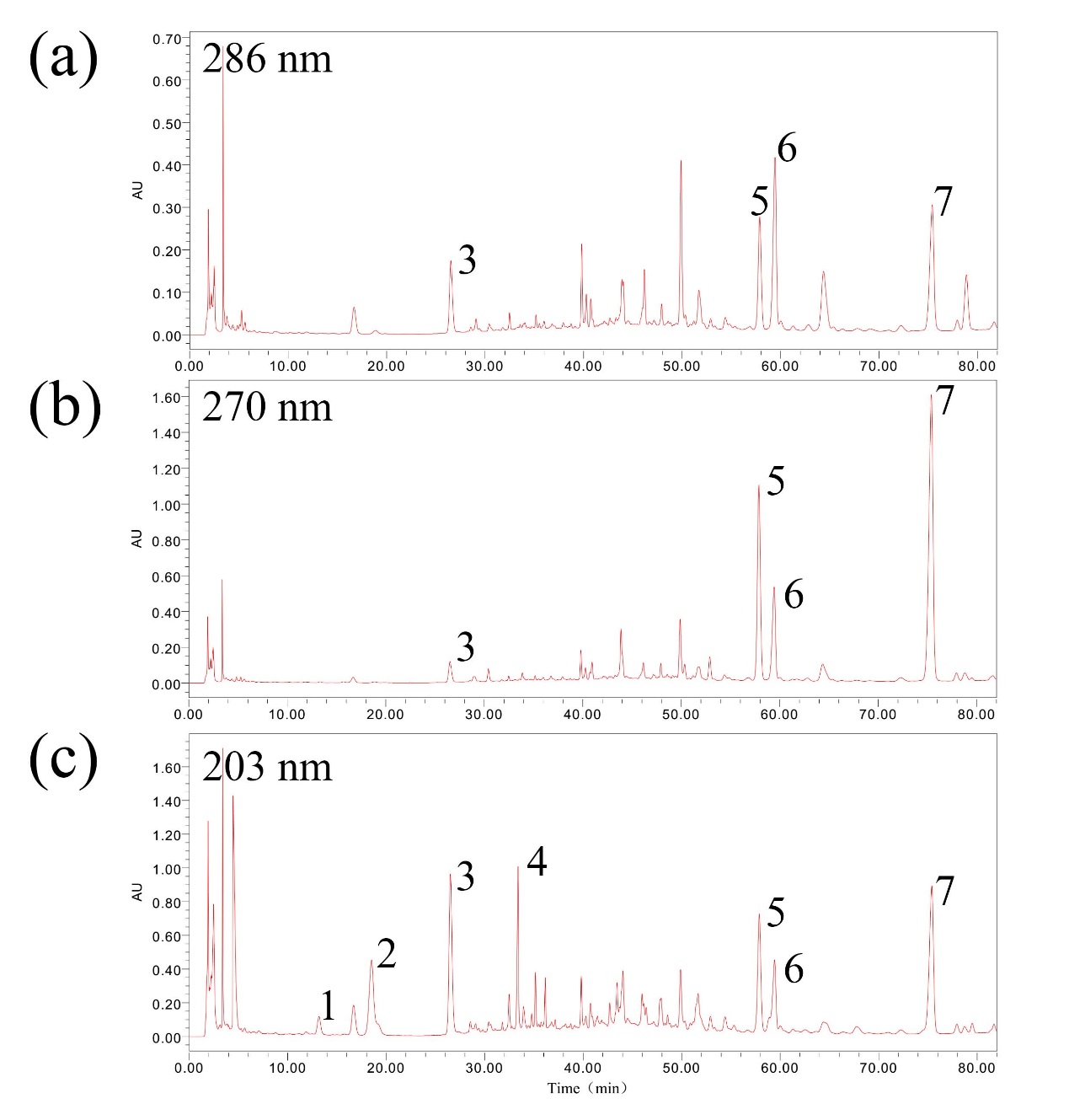


**Supplementary Figure 2. Typical chromatograms of GXDSF in different detection wavelength.** Seven peaks in total were identified and quantified by comparison with reference compounds.


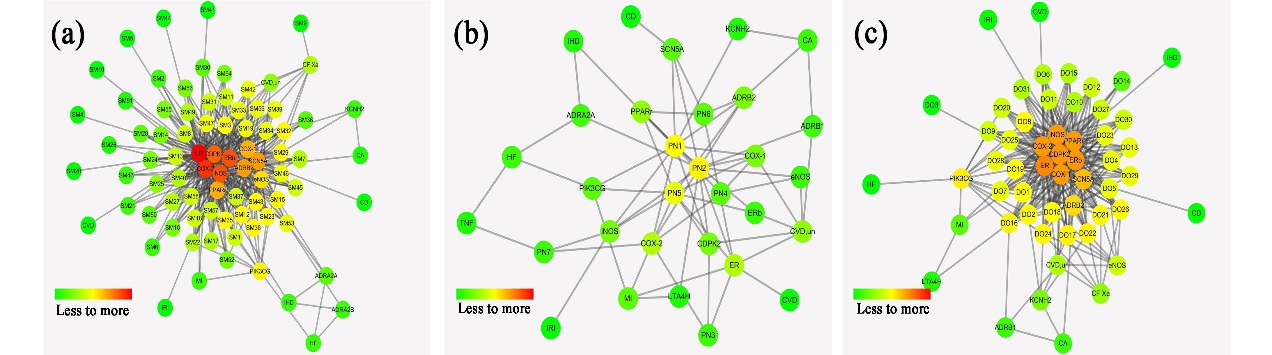


**Supplementary Figure 3. Component-target-CVD networks of Salviae miltiorrhizae Radix et Rhizoma (a), Notoginseng Radix et Rhizoma (b) and the oil extract of Dalbergiae odoriferae Lignum (c).** The networks were analysed using the NetworkAnalyzer plugin in Cytoscape 3.4.0 by mapping the degree parameter as the node, with low values mapped to bright colours, and organized using the Allegro Spring-Electric algorithm. CVD, un, Cardiovascular disease, unspecified; HF, Heart failure; IHD, Ischaemic heart disease; MI, Myocardial infarction; CA, Cardiac arrhythmias; CD, Cardiac dysrhythmias; CVD, Cardiovascular disease; IRI, Ischemia reperfusion injuries.

**
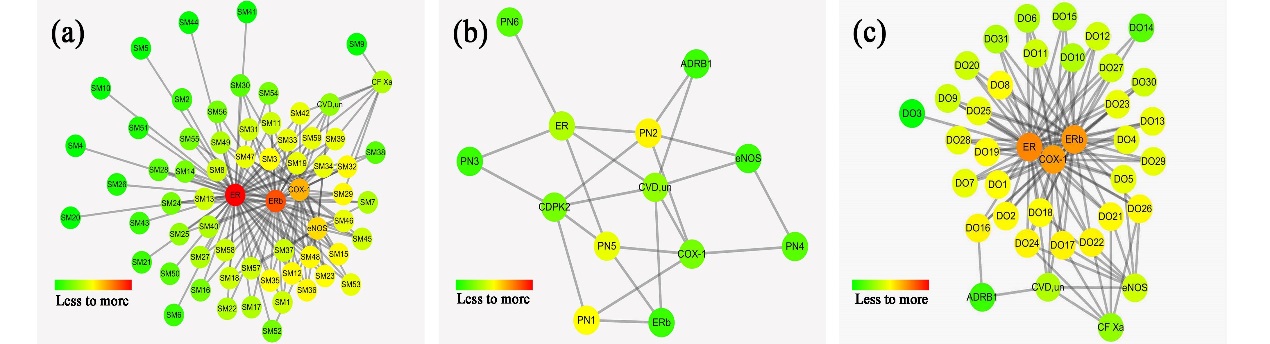
**

**Supplementary Figure 4. Degree-based screening networks of Salviae miltiorrhizae Radix et Rhizoma (a), Notoginseng Radix et Rhizoma (b) and the oil extract of Dalbergiae odoriferae Lignum (c).** The networks were analysed using the NetworkAnalyzer plugin in Cytoscape 3.4.0 by mapping the degree parameter as the node, with low values mapped to bright colours, and organized using the Allegro Spring-Electric algorithm.

1. ^1^Institute of Medicinal Plant Development, Chinese Academy of Medical Sciences and Peking Union Medical College, Beijing, China, ^2^Beijing Key Laboratory of Innovative Drug Discovery of Traditional Chinese Medicine (Natural Medicine) and Translational Medicine, Beijing, China, ^3^Key Laboratory of Efficacy Evaluation of Chinese Medicine against glycerolipid metabolism disorder disease, State Administration of Traditional Chinese Medicine, China, ^4^Zhongguancun Open Laboratory of the Research and Development of Natural Medicine and Health Products, Beijing, China, ^5^Key Laboratory of Bioactive Substances and Resources Utilization of Chinese Herbal Medicine, Ministry of Education, China, ^6^Yunnan Branch, Institute of Medicinal Plant, Chinese Academy of Medical Sciences, Peking Union Medical College, Jinghong Yunnan, China

   ^†^These authors have contributed equally to this work.

   *Corresponding authors should be addressed to Xiaobo Sun (e-mail: sun_xiaobo163@163.com) or Guibo Sun (e-mail: sunguibo@126.com). [↑](#footnote-ref-1)
